# Root-derived carbon and nitrogen from beech and ash trees differentially fuel soil animal food webs of deciduous forests

**DOI:** 10.1371/journal.pone.0189502

**Published:** 2017-12-13

**Authors:** Sarah L. Zieger, Silke Ammerschubert, Andrea Polle, Stefan Scheu

**Affiliations:** 1 University of Göttingen, J.F. Blumenbach Institute of Zoology and Anthropology, Animal Ecology, Göttingen, Germany; 2 University of Göttingen, Büsgen Institute, Forest Botany and Tree Physiology, Göttingen, Germany; 3 University of Göttingen, Centre of Biodiversity and Sustainable Land Use, Göttingen, Germany; University of Copenhagen, DENMARK

## Abstract

Evidence is increasing that soil animal food webs are fueled by root-derived carbon (C) and also by root-derived nitrogen (N). Functioning as link between the above- and belowground system, trees and their species identity are important drivers structuring soil animal communities. A pulse labeling experiment using ^15^N and ^13^C was conducted by exposing beech (*Fagus sylvatica*) and ash (*Fraxinus excelsior*) seedlings to ^13^CO_2_ enriched atmosphere and tree leaves to ^15^N ammonium chloride solution in a plant growth chamber under controlled conditions for 72 h. C and N fluxes into the soil animal food web of beech, associated with ectomycorrhizal fungi (EMF), and ash, associated with arbuscular mycorrhizal fungi (AMF), were investigated at two sampling dates (5 and 20 days after labeling). All of the soil animal taxa studied incorporated root-derived C, while root-derived N was only incorporated into certain taxa. Tree species identity strongly affected C and N incorporation with the incorporation in the beech rhizosphere generally exceeding that in the ash rhizosphere. Incorporation differed little between 5 and 20 days after labeling indicating that both C and N are incorporated quickly into soil animals and are used for tissue formation. Our results suggest that energy and nutrient fluxes in soil food webs depend on the identity of tree species with the differences being associated with different types of mycorrhiza. Further research is needed to prove the generality of these findings and to quantify the flux of plant C and N into soil food webs of forests and other terrestrial ecosystems.

## Introduction

Soil animal communities of deciduous forest form complex food webs [1,2] of high species diversity [3,4]. They comprise a wide spectrum of trophic levels including primary and secondary decomposers, and first, second and third order predators [5,6]. Mesostigmata live as predators [7,8], while Oribatida and Collembola span over a wide range of trophic levels [9,10]. Diplopoda of deciduous forests mainly live as primary decomposers [11]. Soil food webs are connected to plants and the aboveground system via leaf litter input and root-derived resources, with the role of root-derived carbon (C) in fueling soil animal food webs receiving increased attention [12–15]. Further, it has been suggested recently that also root-derived nitrogen (N) contributes to the nutrition of soil animals [16]. Root-derived C and N enter the soil animal food web via living or dead roots, but also via rhizodeposits [17,18]. Rhizodeposits predominantly consist of low molecular weight carbohydrates and therefore are more easily available for soil microorganisms than recalcitrant litter resources [19,20]. Besides the release of C compounds such as sugars, compounds containing both C and N are released by roots such as amino acids and peptides [21]. In particular for N-fixing plants (e.g., legumes) but also non N-fixing plants (e.g., wheat), it has been shown that root-derived N is transferred into the soil, soil microorganisms and neighboring plants [22].

Rhizodeposits, their transformed products and the elements they contain, enter the soil food web via bacteria and fungi, and are transferred to higher trophic levels via bacterial and fungal feeding soil animals, and via predators high up into the food web [12,13,23]. Mycorrhizal and saprotrophic fungi dominate the fungal community of forest soils and play a central role in C and N cycling [24]. Rhizosphere C and N is rapidly taken up by bacteria and fungi with fungi playing a more important role than previously assumed [25,26]. For soil invertebrates, such as Collembola and Oribatida dominating microarthropods in soils, hyphae of saprotrophic and mycorrhizal fungi act as major source of C and nutrients, and their role in animal nutrition likely outweighs that of leaf litter [13,24,27].

One of the most important factors structuring soil animal communities in forests is tree species identity [28,29]. In deciduous forests, tree species associated with ectomycorrhizal fungi (EMF) and arbuscular mycorrhizal fungi (AMF) co-occur. Both mycorrhizal forms release substantial amounts of C into the rhizosphere, but the flux may differ between EMF and AMF [30,31]. Indeed, it has been shown that beech (*Fagus sylvatica*) associated with EMF more intensively affects the rhizosphere as compared to ash (*Fraxinus excelcior*) associated with AMF [28], but the impact of these differences on soil animal nutrition remains elusive.

Due to the small size of most soil animal species and the opaqueness of the soil system, uncovering food relationships via direct observations is limited calling for alternative approaches such as the analysis of natural variations in stable isotope ratios, lipid analysis and molecular gut content analysis [32,33]. In contrast to previous long-term labeling studies investigating the general importance of root-derived C [13,27], we used a pulse (short-term) labeling approach to follow the incorporation of aboveground fixed C and also N into soil animal food webs. Pulse labeling has been used before to follow the incorporation of C into soil animal food webs [34,35], but soil animals rarely have been analyzed at species level and typically only Collembola and Oribatida were investigated [16,36,37]. In a similar experiment conducted in the field, the C label was too low to allow studying differences between tree species [16]. Except for the latter experiment, incorporation of plant N into the soil animal food web has never been studied.

By pulse labeling plants with ^13^C and ^15^N via exposing plant shoots to ^13^CO_2_ enriched atmosphere and via immersing plant leaves into ^15^NH_4_Cl solution [22], we investigated the flux of root-derived C and N into the soil animal food web. Incorporation of ^13^C and ^15^N into soil animal taxa was followed for 5 and 20 days allowing to identify the flux of root-derived C and N into the soil food web. By comparing the flux of C and N into soil animals in the rhizosphere of beech and ash, the role of mycorrhiza type for soil animal nutrition of deciduous forests was investigated.

We hypothesized that (1) incorporation of root-derived C and N into soil animal taxa varies with tree species associated with either EMF (beech) or AMF (ash), with the incorporation in EMF beech exceeding that in AMF ash, and (2) the incorporation rapidly declines with time due to fast turnover rates of root associated microorganisms and microorganism based C and N pools.

## Materials and methods

### Experimental set up

In May 2012 40 beech and 40 ash naturally regenerated young trees (seedlings) were excavated including intact rhizosphere soil, soil biota and litter from a deciduous forest close to the city of Göttingen (51°35'15.39"N 9°58'57.95"E; 360 m asl). The forest consists of 130–145 year old beech trees interspersed with maple and ash. The main soil type is Leptosol with mull humus on limestone and an average pH of ca. 5.3 [3]. Individual seedlings with undisturbed soil were transferred into planting pots of 23 x 23 cm and a depth of 26 cm. Colonization of beech seedlings by EMF was 92.6 ± 2.2% (SE). Colonization of ash by AMF was not measured, but under similar conditions it was 52.6 ± 1.8% (SE). Seedlings were about 1 m in height (ranging between 73 and 177 cm). Seedlings were kept under the canopy of mature beech trees for two months and then transferred to an outdoor greenhouse for ^15^N labeling. The seedlings were irrigated regularly and herbs were removed by cutting the shoots at soil surface level.

### Labeling

The seedlings were labeled in four batches of ten seedlings of the same species each with a time lapse of 12 days between the batches. For ^15^N labeling 98 atom % ^15^N ammonium-chloride (Campro Scientific, Berlin, Germany) was used. Control seedlings were treated with unlabeled ammonium-chloride (Merck, Darmstadt, Germany). In both treatments a 20 mM solution was mixed with sterile distilled water stored at -20°C until usage. At each of three heights of the seedlings (approximately 30, 60 and 90 cm), three leaves of beech and three leaflets of the compound leaf of ash were put in 20 ml scintillation vials containing ammonium-chloride solution for 72 h. To increase label uptake, the leaf surface was abraded with sand paper (Basic Korn 240, LUX, Wermelskirchen, Germany) [38]. The vials were enclosed by using parafilm and placed into plastic bags. To avoid contamination of the soil by leaching of label, planting pots were covered with plastic bags tightened at the stem of the seedlings with Terostat (Teroson Terostat-VII, Henkel, Düsseldorf, Germany). After labeling, leaves and leaflets used for labeling were cut and removed.

After immersion of the leaves into the ^15^N labeling solution, ten seedlings of the same species were transferred to an air-tight plant growth chamber with a surface area of 95 x 114 cm and a height of 200 cm (S2 Fig). Conditions in the chamber were kept at 1,013 hPa, 20°C and 70% relative humidity, light intensity was 420 μE for 16 h per day. An irrigation system consisting of PVC tubes of an inner diameter of 6 mm (Deutsch & Neumann, Berlin, Germany) fixed with cable connection to the plastic bag (OBO Bettermann GmbH & Co. KG, Menden, Germany) of the planting pots were established. A ventilation system was used for measuring soil respiration within the plastic bags. Pipes from the inside of the plastic bag were connected to 1 M sodium hydroxide solution and opened once a day for 30 min to allow free air exchange.

Seedlings were acclimatized for two days at 400 ppm with unlabeled CO_2_ before the ^13^C labeling started by using a 0.5 M solution of sodium carbonate (KMF Laborchemie Handes, Lohmar, Germany). Actual CO_2_ concentration in the chamber was measured using an infrared gas analyzer (CARBOCAP™ Serie GMM220, Driesen + Kern GmbH, Bad Bramstedt, Germany) attached to a regulator connected to a pump system releasing 5 M lactic acid and sodium carbonate into one flask when the CO_2_ concentration fell below 400 ppm. The liberated CO_2_ was pumped into the plant growth chamber. After acclimation seedlings were exposed to ^13^CO_2_ for 3 days for 16 h per day with a maximum CO_2_ concentration of 1800 ppm. For ^13^CO_2_ labeling a total of 2.2 and 3 l 0.5 M sodium carbonate solution with 99 atom% ^13^C sodium carbonate (Sigma-Aldrich, Traufkirchen, Germany) was used for the ten beech and the ten ash seedlings being equivalent to 124 and 155 g sodium carbonate, respectively. To reduce dilution of the ^13^CO_2_ by plant derived CO_2_ at night, CO_2_ in the chamber was absorbed by pumping the air through 1 M sodium hydroxide solution (see S2 Fig).

### Sampling

Five seedlings of each batch were harvested after 5 days, the other five after 20 days after start of the ^13^C labeling, resulting in 10 replicates per tree species and sampling date. Control seedlings were kept in a greenhouse at respective conditions and harvested at the same dates as the labeled seedlings. Litter was collected and soil samples were taken from 0–10 and 10–25 cm soil depth. Animals of the litter and soil were extracted by heat [39] and collected in diethylene glycol-water solution (1:1). Animals were stored at -15°C in 70% ethanol until identification and further processing. Diethylene glycol and ethanol do not affect ^15^N values and only slightly decrease ^13^C signatures [40]. As we used labelling typically resulting in marked changes in ^13^C values and treated all samples in the same way we assume changes due to extraction and storage of animals to be negligible. Fresh fine roots (diameter ≤ 1mm) of beech and ash were sampled after taking soil cores for extracting soil animals. Soil particles were carefully removed from the roots and mycorrhizal root tips were separated from the piece before the tip (lateral root) before samples were freeze-dried, ground in a ball mill (Retsch Schwingmuehle MM400, Retsch GmbH, Haan, Germany) and stored in a desiccator until further analysis.

### Stable isotope analysis

For dual ^13^C and ^15^N measurement 25–612 μg of animal tissue were transferred into tin capsules and dried at 40°C for 48 h; several individuals of small species had to be pooled to obtain enough tissue material for stable isotope analysis. The animals analyzed were taken from soil except for *Tomocerus flavescens*, *Tomocerus vulgaris* and *Steganacarus magnus* which were taken from litter. Stable isotope ratios were analyzed with a coupled system consisting of an elemental analyzer (for samples > 100 μg: NA 2500, CE-Instruments, Rodano, Milan, Italy; for samples < 100 μg: NA1110, CE-Instruments, Rodano, Milan, Italy) and a mass spectrometer (Delta plus, Finnigan MAT, Bremen, Germany) coupled by a ConFlo III interface (Thermo Electron Corporation, Bremen, Germany) [41]. For stable isotope analysis 1–2 mg dry weight of fine roots, mycorrhizal root tip and lateral root were filled into tin capsules and analyzed with a coupled system consisting of an elemental analyzer NA 1108, Fisons-Instruments, Rodano, Milan, Italy and a mass spectrometer (Delta C, Finnigan MAT, Bremen, Germany) coupled by a ConFlo III interface (Thermo Electron Corporation, Bremen, Germany). Abundances of ^13^C and ^15^N are expressed using the δ notation with δ_sample_ [‰] = [(R_sample_ − R_standard_)/R_standard_] × 1000; R_sample_ and R_standard_ represent the ^13^C/^12^C and ^15^N/^14^N ratios of samples and standard, respectively. For ^13^C PD Belemnite (PBD) and for ^15^N atmospheric nitrogen served as the primary standard. Acetanilide (C_8_H_9_NO, Merck) was used for internal calibration.

For analyzing the enrichment in ^13^C and ^15^N of soil animal taxa we calculated the difference in delta values between animals from labeled and unlabeled trees as Δ_element_ = δ_label_—δ_control_, with Δ_element_ representing the Δ^13^C and Δ^15^N values. Samples with mean Δ^13^C and Δ^15^N in the range of two standard deviations of δ^13^C and δ^15^N of respective control samples were assumed not to be enriched and set to zero.

### Statistical analysis

Statistical analyses were performed using R v.3.2.4 (R Core Team 2016). Δ^15^N and Δ^13^C values of eleven soil animal taxa were analyzed separately using linear mixed effects models [42]. A random effect of tree identity (tree ID) avoiding pseudo-replication of soil animal taxa of the same tree was included. We tested the effect of Tree species (beech and ash) and Sampling date (5 and 20 days after labeling) and their interactions on C and N incorporation into soil animal taxa. Δ^15^N and Δ^13^C of the two root compartments (lateral root and mycorrhizal root tip) were analyzed separately using linear mixed effects models. A random effect of root tip identity (root ID) avoiding pseudo-replication of compartments of the same root tip was included. We tested the effect of compartment (lateral root and mycorrhizal root tip) and Sampling date (5 and 20 days after labeling) and their interactions on C and N incorporation into lateral root and mycorrhizal root tip. Δ^15^N and Δ^13^C values were log-transformed to improve homogeneity of variance. The model was simplified by stepwise reduction ending up with two models, one for enrichment in C and one for enrichment in N. To inspect differences between animal species for C and N enrichment in beech and ash contrast analyses were performed testing differences within each type of Tree species. Further, individual analyses of variance were performed for each species (see S3 Table). Linear mixed effects models with the intercept set to zero were used to inspect C and N enrichment in animal taxa to be significantly different from zero within each level of Tree species. Data given in text represent means and standard deviations.

## Results

### Natural abundance

Natural abundance δ^13^C signatures of soil animal taxa spanned over 5.06 delta units from -25.33 ± 0.4 ‰ in *Nothrus palustris* (Oribatida) to -20.27 ± 0.73 ‰ in *S*. *magnus* (Oribatida), while natural δ^15^N signatures spanned over 7.44 delta units from -3.00 ± 0.77 ‰ in *N*. *palustris* to 4.44 ± 1.34 ‰ in Onychiuridae (Collembola) (S1 Fig).

### Carbon

**Fig 1:**
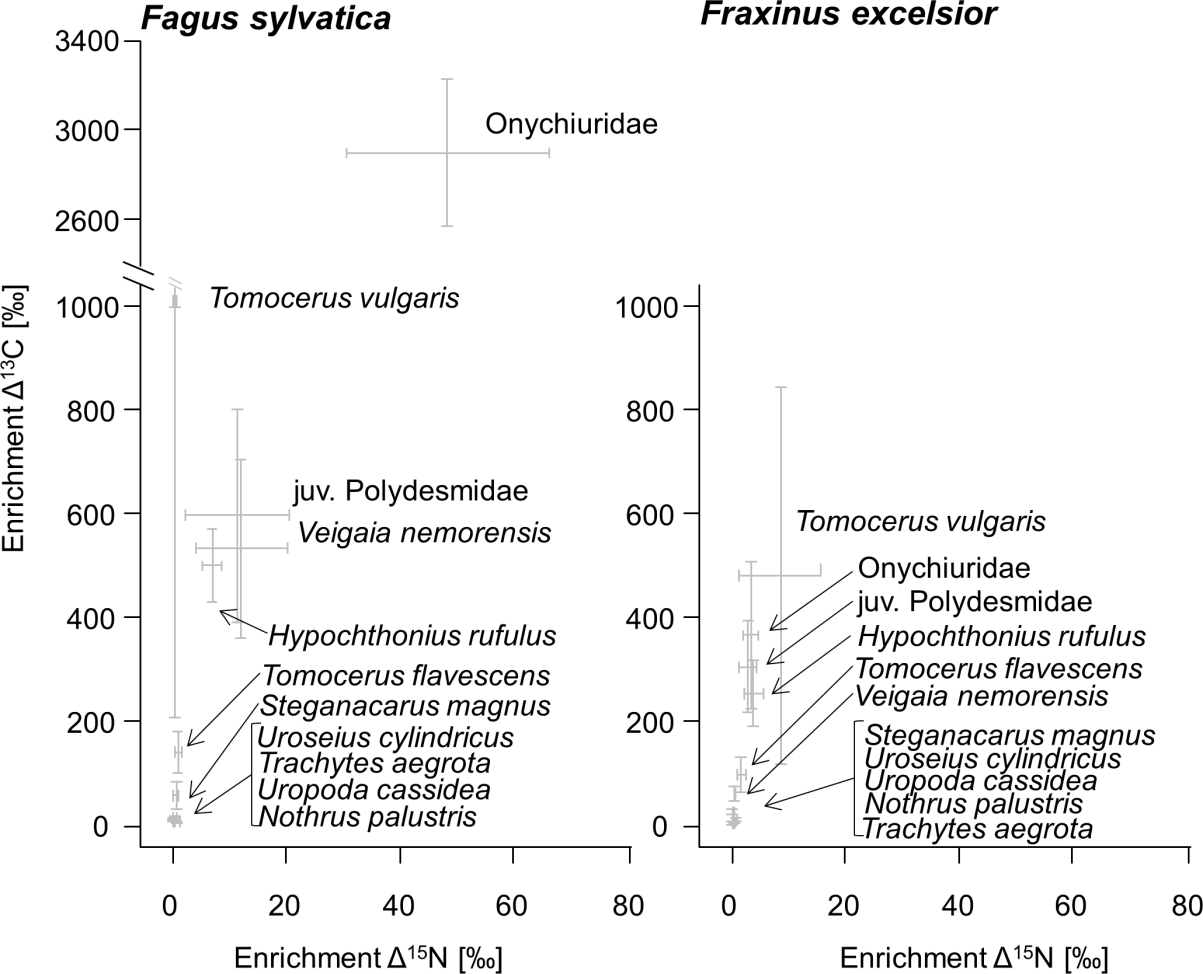
Enrichment in ^15^N and ^13^C in soil animal species / taxa. Enrichment in ^15^N and ^13^C in soil animal species / taxa in the rhizosphere of beech (*Fagus sylvatica*) and ash (*Fraxinus excelsior*). Means and standard error (SE). Note the axis break on the y-axis between 1000 and 2600 ‰.

Each of the 11 analyzed soil animal taxa was enriched in ^13^C (S1 Table) with Δ^13^C values ranging between 0.75 and 4199 ‰, and differing significantly between species (F_10,67_ = 84.39, p < 0.001). Incorporation of root-derived C declined in the order Onychiuridae (1748 ± 1456 ‰) > *T*. *vulgaris* (722.1 ± 1300 ‰) > juvenile Polydesmidae (451.5 ± 398.0 ‰) > *Hypochthonius rufulus* (377.2 ± 202.1 ‰) > *Veigaia nemorensis* (319.2 ± 386.3 ‰) > *T*. *flavescens* (120.6 ± 89.56 ‰) > *S*. *magnus* (43.45 ± 48.50 ‰) > *Uroseius cylindricus* (13.53 ± 8.65 ‰) > *Trachytes aegrota* (9.48 ± 5.14 ‰) > *Uropoda cassidea* (8.23 ± 3.81 ‰) > *N*. *palustris* (6.96 ± 5.06 ‰). The incorporation differed between tree species (significant Animal taxa × Tree species interaction; F_10,67_ = 4.46, p = < 0.001, S2 Table), but soil animal taxa generally were more enriched in the beech as compared to the ash rhizosphere (Fig 1), which was mainly due to *H*. *rufulus*, Onychiuridae, *T*. *aegrota* and *V*. *nemorensis* (see individual analysis of variance S3 Table). Animal taxa differentially incorporated root-derived C at the two sampling dates (significant Animal taxa × Sampling date interaction; F_10,67_ = 3.68, p = 0.001), which was mainly due to significant higher enrichment at day 20 after labeling in *U*. *cassidea* (10.58 ± 1.28 ‰) as compared to 5 days after labeling (5.88 ± 1.22 ‰). Enrichment in ^13^C in fine roots of ash exceeded that in beech but differences were only marginally significant (3163 ± 1121 and 2341 ± 1034 ‰ respectively; F_1,20_ = 3.52, p = 0.075). In beech, ectomycorrhizal root tips were significantly more enriched in ^13^C (3771 ± 4514 Δ ‰) than lateral roots (2428 ± 2380 Δ ‰, F_1,32_ = 6.52, p = 0.016).

### Nitrogen

Six of the 11 analyzed soil animal taxa were significantly enriched in ^15^N with Δ^15^N values (S1 Table) ranging between zero and 121.0 ‰, and differing significantly between species (F_10,77_ = 7.55, p < 0.001). Incorporation of root-derived N declined in the order Onychiuridae (27.93 ± 38.92 ‰) > juv. Polydesmidae (7.15 ± 16.04 ‰) > *V*. *nemorensis* (6.83 ± 15.39 ‰) > *H*. *rufulus* (5.42 ± 4.26 ‰) > *T*. *vulgaris* (4.83 ± 12.03 ‰) > *T*. *flavescens* (1.27 ± 1.72 ‰) > *U*. *cylindricus* (0.60 ± 0.62 ‰) > *N*. *palustris* (0.47 ± 0.77 ‰) > *S*. *magnus* (0.42 ± 0.83 ‰) > *U*. *cassidea* (0.35 ± 0.47 ‰) > *T*. *aegrota* (0.22 ± 0.33 ‰). However, the incorporation differed between tree species (significant Animal taxa × Tree species interaction; F_10,77_ = 2.90, p = 0.004, S2 Table), which was mainly due to significant higher incorporation in Onychiuridae in the beech as compared to the ash rhizosphere (F_1,11_ = 5.37, p = 0.046; Fig 1). Incorporation of root-derived N did not vary with sampling date (F_1,29_ = 0.003, p = 0.952) and therefore sampling date was removed from the final model. Enrichment in ^15^N in fine roots of beech and ash did not differ significantly (F_1,20_ = 0.28, p = 0.59) being on average 181.9 ± 130.3 ‰. In contrast to ^13^C, ectomycorrhizal root tips of beech were significantly less enriched in ^15^N (51.26 ± 76.67 Δ ‰) as compared to lateral roots (134.36 ± 144.49 Δ ‰, F_1,32_ = 46.21, p = < 0.001).

## Discussion

### Natural abundance

The classification of the studied soil animal taxa into trophic groups according to natural variations in δ^15^N signatures resembled earlier findings [6,8–10,43]. High δ^15^N signatures in Onychiuridae (Collembola) has been reported previously [10,44], however, rather than living on animal prey high ^15^N signatures may also indicate feeding on mycorrhiza [45] as mycorrhiza sheaths of root tips are enriched in ^15^N [46].

### Incorporation of root-derived C

Each of the analyzed soil animal taxa incorporated root-derived C shortly after labeling indicating that recently plant assimilated C is rapidly transferred into the soil animal food web of beech and ash trees. This reinforces the importance of root-derived C in fueling soil animal food webs [13,15,27]. However, incorporation of root-derived C varied markedly between soil animal taxa as shown previously [15,27]. Under beech, Onychiuridae were most enriched in ^13^C and ^15^N with the ^13^C enrichment exceeding that in fine roots indicating that they fed on root and / or mycorrhizal tissue more enriched in ^13^C than bulk root tissue. Indeed, mycorrhiza were more enriched in ^13^C as compared to roots reflecting that recently assimilated C is rapidly transferred to mycorrhiza [47,48]. Supporting the conclusion that Onychiuridae fed on roots and / or mycorrhizal tissue, it has been shown previously that Onychiuridae feed on roots, however, this has only been shown for herbaceous plants [49]. Further, in food choice experiments it has been shown that Collembola feed on mycorrhizal fungi [50]. In line with these findings Collembola have been shown to rapidly incorporate root-derived C in field experiments [34,51–53], with high amounts being incorporated into Onychiuridae [36,37]. Also, the fast tissue turnover of Collembola likely contributed to the high incorporation of root-derived C in Onychiuridae, as previously suggested [34].

In contrast to Onychiuridae, ^13^C enrichment in juvenile Polydesmidae did not exceed that in fine roots, but they were also highly enriched in ^13^C and ^15^N indicating that they also feed on root material. Indeed, it has been shown previously that Polydesmidae feed on root hairs [54] and there is evidence that mixed diets of fungi and plant material increase fertility and growth of Polydesmidae [55]. However, in a root labeling experiment Diplopoda were only moderately enriched in ^13^C suggesting that they predominantly feed on other resources [14]. As Diplopoda including Polydesmidae are strongly sclerotized slow tissue turnover may have contributed to lower incorporation of root-derived C as compared to the less sclerotized root feeders.

The two predacious mite species studied, *H*. *rufulus* (Oribatida) and *V*. *nemorensis* (Gamasina), also were highly enriched in ^13^C indicating that they heavily rely on root resources presumably via feeding on Nematoda or Collembola relying on root-derived C. Indeed, *V*. *nemorensis* [7,56] and *H*. *rufulus* [57,58] are known to feed on Collembola including Onychiuridae [59]. Further, there is increasing evidence that Gamasina heavily rely on Nematoda prey [60], but see also Kudrin et al. [61].

In the two analyzed *Tomocerus* species the enrichment in ^13^C was intermediate, indicating that to some extent root-derived resources contribute to their diet, which is in line with previous studies [36,37]. Low incorporation of root-derived N suggests that it was not based on root feeding but rather on feeding on rhizosphere microorganisms which had incorporated N from root exudates. *Tomocerus* species are known to feed on a variety of food materials including litter but also fungi and bacteria in the rhizosphere [62], algae [63] as well as Nematoda [64]. Omnivory in Collembola has been proposed previously [65] and mixed diets have been shown to increase growth in Collembola [66]. The high variance in the natural abundance of stable isotopes found in the present study also indicate that the diet of *Tomocerus* species varies between individuals. Overall, incorporation of root-derived C but little root-derived N likely was due to feeding on mycorrhizal fungi but the intermediate ^13^C signature suggests that they only form a small fraction of the diet of *Tomocerus* species.

The Uropodina species studied, *U*. *cassidea*, *U*. *cylindricus* and *T*. *aegrota*, incorporated little root-derived C. Uropodina are slow moving mites which are assumed to feed on Nematoda [64,67,68]. However, at least *T*. *aegrota* also has been suggested to feed on fungi [6,69]. The low contribution of root-derived C indicate that their prey relies little on root-derived resources. However, in the long-term *U*. *cassidea* has been shown to rely on root-derived C [13]. Presumably, slow tissue turnover contributed to the low incorporation of root-derived C in the present study.

Similar to Uropodina, the Oribatida species *N*. *palustris and S*. *magnus* were little enriched in ^13^C indicating that they rely little on root-derived resources. This is consistent with their trophic position as primary decomposers suggesting that they predominantly feed on dead organic matter such as leaf litter [9,70]. Higher incorporation of root-derived C in *S*. *magnus* than in *N*. *palustris* suggests differential incorporation of resources supporting earlier findings on niche differentiation in Oribatida [6,9].

### Incorporation of root-derived N

Six of the eleven analyzed soil animal taxa incorporated root-derived N indicating that N in plant leaves also is rapidly transferred to the roots and into the soil animal food web. Although this pathway of N received little attention until today (but see e.g., Brumme et al. [71]), it has been shown recently that root-derived N contributes to the nutrition of soil animals in the field [16]. Notably, incorporation of root-derived N varied significantly between soil animal taxa and, in contrast to C, some species did not incorporate any. Presumably, this is due to the fact that rhizodeposits predominantly consist of C rather than N compounds [72]. However, two secondary decomposers, i.e. juvenile Polydesmidae (Diplopoda) and Onychiuridae (Collembola) incorporated root-derived N presumably via feeding on roots and / or root hairs (see above), supporting our conclusion that they heavily rely on root-derived resources.

### Variations with time

As in earlier studies [34], incorporation of root-derived C into soil animal taxa varied with time. However, in contrast to our second hypothesis changes in time were low indicating that the assimilated C was rapidly transported into roots, entered the soil animal food web and stayed there for at least 20 days. Contrasting the general pattern, variation in the incorporation of ^13^C with time was pronounced in *U*. *cassidea* incorporating more C after 20 days than after 5 days, indicating that the prey of this predatory mite species only slowly incorporated root-derived C. Incorporation of root-derived N generally did not vary significantly with time suggesting that N leakage and incorporation into the soil food web stayed rather constant even after the addition of tracer was terminated.

### Variations with tree species

The incorporation of root-derived C into the soil animal food web significantly differed between tree species with the incorporation in the beech rhizosphere exceeding that in the ash rhizosphere. This contrasts results of a long-term experiment in which no difference in the flux of root-derived C from beech and ash into soil animals was found after five months [27]. This indicates that in the long-term the low flux of root-derived resources from ash into the soil animal food web is compensated by the provisioning of other root-derived resources, potentially dead roots. The differential incorporation of C with tree species in the present experiment indicates that the resources provided by roots for fueling rhizosphere food webs vary with time. Presumably, differences between tree species in the provisioning of root-derived resources are related to differences in root morphology [73] or the different types of mycorrhiza in beech and ash with the former being associated with EMF and the latter with AMF [31,74]. Selective feeding on EMF has been found for the Protura *Acerentomon* sp. (S. Zieger, unpubl. data), whereas the species investigated in this study likely fed on mixed diets of root-derived and other resources, which is widespread in soil animals [6,66,75]. However, the high enrichment in ^13^C and ^15^N in Onychiuridae in the beech but not in the ash rhizosphere indicates that Onychiuridae feed on EMF or roots associated with EMF. In fact EMF typically forms a dense mantle around roots and likely is ingested by species feeding on roots. The predatory mites *H*. *rufulus*, *T*. *aegrota* and *V*. *nemorensis* also incorporated more ^13^C in the beech as compared to the ash rhizosphere suggesting that they selectively feed on EMF associated prey species, potentially Onychiuridae and root associated Nematoda.

### Conclusions

All soil animal species studied incorporated root-derived C supporting earlier findings that root-derived C plays an important role in fueling soil animal food webs. Notably, not only root-derived C, but also root-derived N was incorporated into the soil animal food web indicating that plant N contributes to the nutrition of soil animals thereby alleviating N deficiency in soil animal food webs. However, in contrast to C not all animal species incorporated root-derived N, presumably as rhizodeposits predominantly consist of C rather than N compounds. Differential incorporation of root C and N suggests that root resources contribute to niche differentiation in soil animal species. Incorporation of root C, but not root N, varied with time suggesting that root-derived C not only contributes to animal metabolism, but to animal body tissue formation and quickly is propagated from low to high trophic levels. Notably, incorporation of root-derived C and N into soil animals varied with tree species, i.e. between beech and ash, indicating that tree identity and mycorrhizal type plays an important role in fueling soil animal food webs. Overall, the results underline the importance of root-derived resources in fueling soil animal food webs and suggest that this not only applies to C but also to N.

## Supporting information

S1 Fig Natural abundance stable isotope signatures. Natural abundance of δ^15^N and δ^13^C signatures of the soil animal species / taxa investigated. Means and standard error (SE) with numbers of replicates in brackets.
(PPTX)

S2 Fig Experimental set up. **A:** Soil respiration was measured for each of the ten seedlings separately; **B:** Night filter, to reduce dilution of the applied ^13^CO_2_ by plant-derived CO_2_ at night.
(TIF)

S1 Table Enrichment analysis. Estimate, standard error (SE), t-value and p-value of linear mixed effects models with the intercept set to zero on the enrichment in Δ^13^C and Δ^15^N in animal taxa within each level of Tree species (beech and ash).
(XLSX)

S2 Table Contrast analysis. Estimate, standard error (SE), z-value and p-value of contrast analysis of differences in Δ^13^C and Δ^15^N values between animal taxa in the rhizosphere of beech and ash (Δ ^13^C only); full contrast matrix of linear mixed effects models.
(XLSX)

S3 Table Individual ANOVA. F- and p-values of the effect of Tree species (beech and ash) and Sampling after labeling (5 and 20 days) on the enrichment in ^13^C in animal taxa and the effect of Tree species (beech and ash) on the enrichment in ^15^N.
(XLSX)

S4 Table Dataset. ^13^C and ^15^N stable isotope values of soil animal taxa (Animal taxa) from soil or litter samples (Layer) of control and labeled (Treatment) beech and ash trees (Tree species) sampled after 5 and 20 days (sampling date) after ^13^C labeling.
(XLSX)
