# Supplementary material for: Root-derived carbon and nitrogen from beech and ash trees differentially fuel soil animal food webs of deciduous forests
Source: PLoS One. 2017 Dec 13;12(12):e0189502. doi: 10.1371/journal.pone.0189502 (PMC5728517; doi:10.1371/journal.pone.0189502)
Supplement: S1 Fig — Natural abundance of δ15N and δ13C signatures of the soil animal species / taxa investigated. Means and standard error (SE) with numbers of replicates in brackets. (PPTX) [file pone.0189502.s001.pptx]

## Slide 1
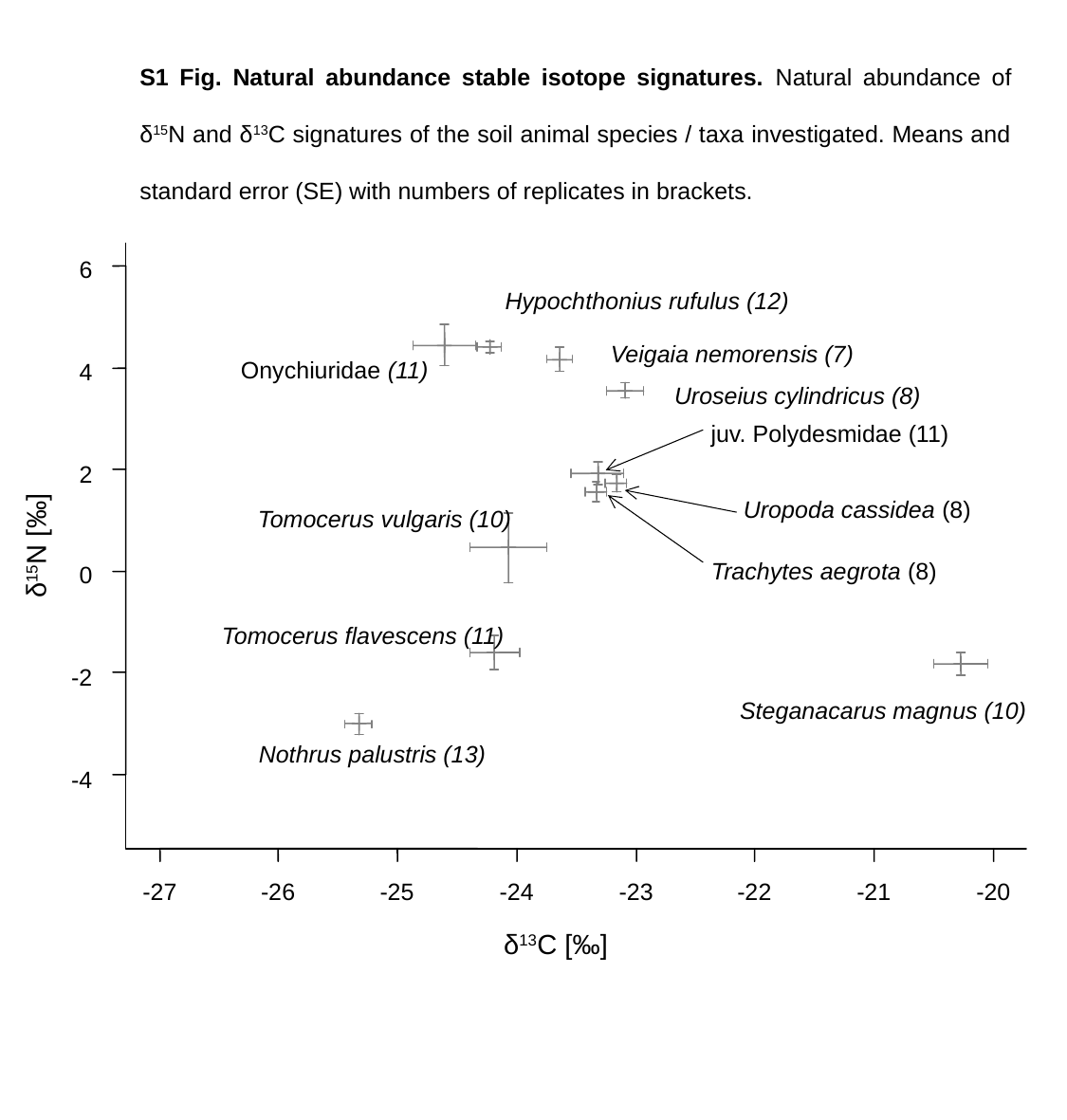

S1 Fig. Natural abundance stable isotope signatures. Natural abundance of δ15N and δ13C signatures of the soil animal species / taxa investigated. Means and standard error (SE) with numbers of replicates in brackets.
6
Hypochthonius rufulus (12)
Veigaia nemorensis (7)
Onychiuridae (11)
4
Uroseius cylindricus (8)
juv. Polydesmidae (11)
2
Uropoda cassidea (8)
Tomocerus vulgaris (10)
δ15N [‰]
Trachytes aegrota (8)
0
Tomocerus flavescens (11)
-2
Steganacarus magnus (10)
Nothrus palustris (13)
-4
-27
-26
-25
-24
-23
-22
-21
-20
δ13C [‰]
