# Supplementary figures and images for: Root-derived carbon and nitrogen from beech and ash trees differentially fuel soil animal food webs of deciduous forests
Source: PLoS One. 2017 Dec 13;12(12):e0189502. doi: 10.1371/journal.pone.0189502 (PMC5728517; doi:10.1371/journal.pone.0189502)

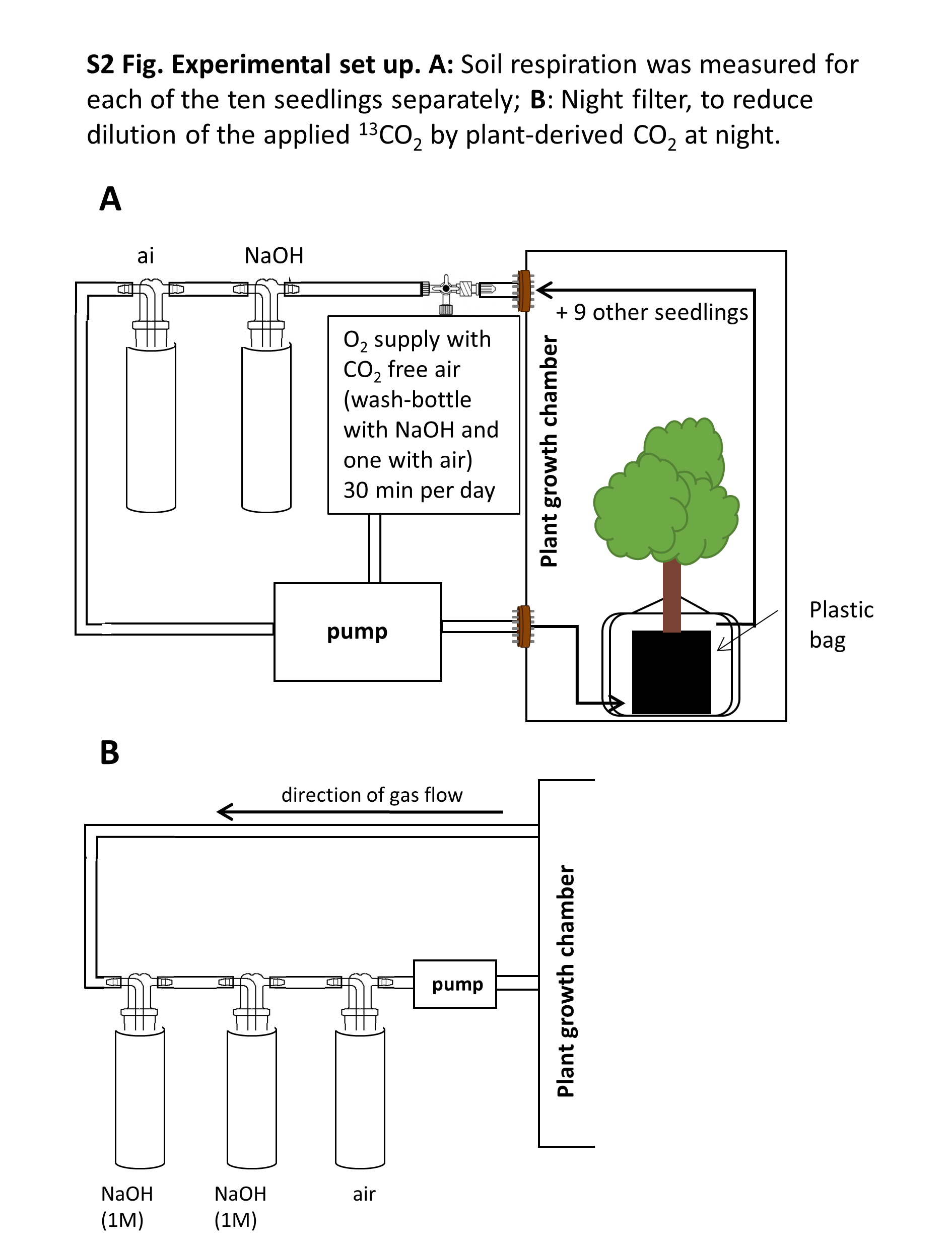

Supplement: S2 Fig — A: Soil respiration was measured for each of the ten seedlings separately; B: Night filter, to reduce dilution of the applied 13CO2 by plant-derived CO2 at night. (TIF) [file pone.0189502.s002.tif]
